# MS1FA: Shiny app for the annotation of redundant features in untargeted metabolomics datasets

**DOI:** 10.1093/bioinformatics/btaf161

**Published:** 2025-04-15

**Authors:** Ruibing Shi, Frank Klawonn, Mark Brönstrup, Raimo Franke

**Affiliations:** Biostatistics Research Group, Helmholtz Centre for Infection Research, Braunschweig, 38124, Germany; Biostatistics Research Group, Helmholtz Centre for Infection Research, Braunschweig, 38124, Germany; Department of Computer Science, Ostfalia University of Applied Sciences, Wolfenbüttel, 38302, Germany; Department of Chemical Biology, Helmholtz Centre for Infection Research, Braunschweig, 38124, Germany; German Center for Infection Research (DZIF), Partner Site Hannover-Braunschweig, Braunschweig, 38124, Germany; Institute of Organic Chemistry, Leibniz University Hannover, Hannover, 30167, Germany; Department of Chemical Biology, Helmholtz Centre for Infection Research, Braunschweig, 38124, Germany

## Abstract

**Motivation:**

Untargeted metabolomics, the comprehensive analysis of small molecules in biological systems, has become an invaluable tool for understanding physiology and metabolism. However, the annotation of metabolomic data is often confounded by the presence of redundant features, which can arise from e.g. multimerization, in-source fragments (ISFs), and adducts.

**Results:**

MS1FA uniquely integrates all major annotation approaches for redundant features within a single interactive platform. It combines correlation-based grouping with reliable ISF annotation using MS2 data and operates with MS1 data only, MS2 data only, or both. Additionally, it offers a distinctive method for grouping features based on relational criteria. As the only web-based platform with these capabilities, MS1FA provides easy access and allows users to explore and annotate the feature table interactively, with options to download the results.

**Availability and implementation:**

MS1FA is freely accessible at https://ms1fa.helmholtz-hzi.de. The source code and data are available at https://github.com/RuibingS/MS1FA_RShiny_dashboard and are archived with the DOI 10.5281/zenodo.15118962.

## 1 Introduction

Untargeted metabolomics aims to identify and quantify all metabolites in a biological sample. Liquid chromatography coupled with electrospray ionization (ESI) mass spectrometry is the most common analytical method used for this purpose. It offers versatile and sensitive detection of metabolites, but still poses the challenge to annotate tens of thousands of features and to identify metabolites. The majority of peaks in untargeted LC-MS based metabolomics datasets correspond to isotopes, redundant ions caused by ionization-related phenomena, such as adduct formation, multimerization and in source-fragmentation (ISF), or contaminants and MS or bioinformatics artifacts. A correct and comprehensive annotation of redundant features is crucial for accurate and reliable metabolite identification. Simply removing isotopes, adducts, and in-source fragments (ISFs) does not inherently resolve this issue. Instead, these features contain essential structural and ionization information that, when systematically integrated, improve annotation accuracy rather than hinder it. Clark *et al.* demonstrated that depending on the mass spectrometer used, differences in fragmentation, charge states and adduct formation can cause substantial differences and only 25% overlap between the feature tables of the same samples (Clark *et al.* 2021). Xu *et al.* found a high number of cases where in-source fragments mimic cellular metabolites which can result in misannotation and misidentification, potentially leading to wrong conclusions (Xu *et al.* 2015).

Various tools have been developed to aid feature annotation and address the problem of degenerate features. CAMERA, one of the earliest available tools, uses peak shape correlation to group features and annotate isotopes, adducts and typical neutral losses (Kuhl *et al.* 2012). A similar approach is followed by the R package CliqueMS (Senan *et al.* 2019). Ion identity molecular networking (IIMN) developed in the Dorrestein group integrates peak shape correlation analysis into molecular networking analysis (Schmid *et al.* 2021). NetID uses a global network optimization to annotate peaks based on biochemical transformations (Chen *et al.* 2021). ISFrag uses MS2 data to identify in-source fragments in LC-MS full scan data (Guo *et al.* 2021). However, it is still a major challenge to recognize all the redundant features and correctly annotate them.

Here, we introduce the novel tool MS1FA (MS1 feature annotation), which we designed to combine multiple approaches within one platform and offer the possibility to work with only MS1 data, with MS1 data combined with a pool sample measured in MS2 mode, or only MS2 files.

## 2 Materials and methods

**Figure 1.**
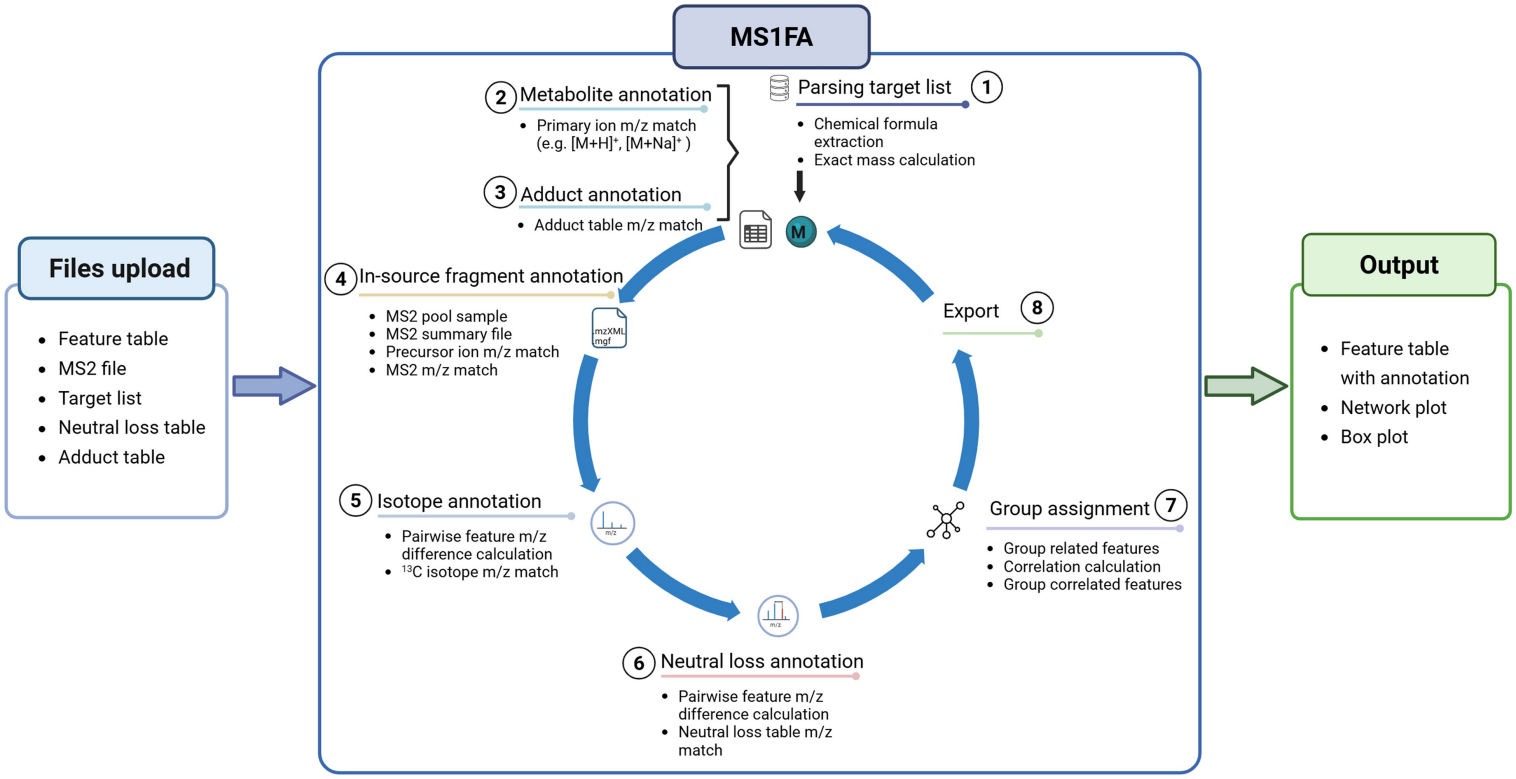
The MS1FA workflow, consisting of three main steps: file upload (left panel), MS1FA algorithm (center panel), and output generation, including the annotated feature table and plots (right panel). Created with BioRender.com.

MS1FA was developed in R and implemented as Shiny app, with essential parts written in C++ using the RCPP framework for fast processing and computation. It runs on a Shiny server which is freely accessible via https://ms1fa.helmholtz-hzi.de. The source code is deposited as Github archive: https://github.com/RuibingS/MS1FA_RShiny_dashboard. Figure 1 shows the workflow and processing steps of MS1FA which are discussed in the following paragraph.

### 2.1 MS1FA inputs

A feature table generated by a peak picking algorithm such as XCMS or MZmine has to be uploaded (Fig. 1, left). To make use of the ISF annotation, MS2 data have to be provided, which can be either in the form of a single mzXML or mzML file from a pool sample or a mgf-file which can be generated from multiple files by MZmine. For metabolite annotation, a target list needs to be uploaded. MS1FA includes a predefined neutral loss table compiled by Oliver Fiehn’s lab (Ma *et al.* 2014) and a default ESI-MS adduct table assembled from published lists (Kuhl *et al.* 2012, Ma *et al.* 2014), but the users can also upload their own neutral loss and adduct annotation tables.

### 2.2 MS1FA algorithm

The steps of the MS1FA algorithm are depicted in Fig. 1, center:

1. The target list, containing the names and molecular formulas of metabolites to be annotated, is parsed and primary ions are calculated from the molecular formulas. If there is no target list available, the workflow can still be executed without one.
2. The calculated primary ions are matched against the uploaded feature table by exact mass (*m*/*z*) and by retention time (rt) if available. The default *m*/*z* tolerance is 0.002 or 5 ppm and the default rt window is 20 s.
3. Adduct annotation is carried out by calculating the adduct *m*/*z* values derived from the primary ion identified in step 2 according to the default common ESI adduct table. Next, adduct features are annotated by matching these *m*/*z* values to those in the feature table. The default *m*/*z* tolerance is 0.005 or 5 ppm and the default rt window is 2 s.
4. In-source fragment (ISF) annotation is achieved by using MS2 data, ideally from data-dependent acquisition (DDA) runs (Supplementary Fig. S2). If DDA MS2 data from a pool sample are uploaded, the precursor ion (PI) *m*/*z* values are first aligned to the feature table *m*/*z* values (derived from MS1 spectra) using a default *m*/*z* tolerance of 0.002 or 5 ppm as threshold along within a default rt window of 20 s. When a PI is matched to a feature in the MS1 feature table, the corresponding MS2 spectrum *m*/*z* values are matched to MS1 features within a default *m*/*z* tolerance of 0.005 or 10 ppm.
5. Annotation of isotope peaks and charge states: A pairwise *m*/*z* difference matrix is constructed within a default rt window of 3 s. ^13^C isotopes are detected by identifying *m*/*z* differences equal to 1.0034 and multiples (up to 4). For the first isotope, *m*/*z* tolerance of 0.002 is set and for the second and higher isotopes threshold 0.01 is used to also capture peaks dominated by heavier isotopes of S, Cl, or Br. To annotate multiply charged ions, *m*/*z* differences of 1.0034, 0.5017, and 0.3345 are detected to assign singly, doubly, and triply charged ions respectively.
6. Neutral loss annotation: MS1FA calculates the *m*/*z* differences for each feature pair in the feature table and compares these against the neutral loss table. Only if a match occurs within a default rt window of 2 s and *m*/*z* tolerance within 0.002 *m*/*z* or 5 ppm, a neutral loss gets annotated.
7. Grouping of features and group index assignment: To identify features derived from the same metabolite or otherwise related features, we categorize them into groups by using two grouping approaches. The first grouping method merges all features that are related to each other, such as in-source fragments, adducts, neutral losses, and isotopes, into one group (Supplementary Information S2.3). The second grouping method is based on perturbation profile similarity (Supplementary Information S2.1 and Supplementary Fig. S1). The basic idea is that if a metabolite has an *x*-fold altered intensity in condition A versus condition B, all features corresponding to this metabolite should have an *x*-fold altered intensity. A Pearson correlation matrix is calculated using log_10_ transformed intensity values with an rt threshold of 2 s and a correlation coefficient threshold of 0.8. A fully connected correlation network plot is constructed, ensuring that any pair of features within “corgroup” has a correlation value exceeding the threshold.

The user can adjust all thresholds and parameters mentioned above, select the desired correlation method (Pearson, Spearman, or Kendall) and interactively inspect the correlation test statistics for any pair of rows.

### 2.3 MS1FA outputs

The output of MS1FA (Fig. 1, right) includes an interactive feature table with all annotations, an interactive correlation network plot generated from the perturbation profile similarity approach, and box plots showing the intensity distribution for the correlated features. Selecting a row in the feature table automatically generates a network plot for the selected feature, where nodes represent feature names, *m*/*z* values, retention times, and annotations, while edges depict the correlation coefficients, *m*/*z* differences and neutral loss annotation if available. Box plots are then generated for these correlated features, illustrating the log_10_ intensity values across the selected samples. The feature table can be filtered in various ways and users can add their own annotations.

## 3 Results

The application of MS1FA was demonstrated using two LC-MS untargeted metabolomics datasets, both provided as demo data within the Shiny application and detailed in the user manual in Supplementary Information S4. The dataset “PA14” was generated by an untargeted metabolomics study of the bacterial pathogen *Pseudomonas aeruginosa* PA14 perturbed by different antibiotics (Franke *et al.* 2021). In an approach we coined “annotation by perturbation,” we generated differential abundances of PA14 metabolites. We use the correlation of feature abundance patterns to group features together in a so-called “corgroup” that should ideally contain only features from the same metabolite. Even without the availability of MS2 data, a deep annotation of features to metabolites of the target list is possible. Supplementary Fig. S3 shows the output for the metabolite phenylalanine with correct grouping of all features by correlation.

We established a second grouping approach, which uses any relationship between features (ISF-MS2 match, adduct, neutral loss, etc.) to link all related features. For instance for the natural product phlorizin, a glucoside of phloretin, MS1FA grouped twenty-seven features via ISF-MS2 relations, adduct-, neutral loss-, and isotope annotation. All features were correctly annotated, and the annotation of the neutral loss as anhydrohexose even provided structural information (Supplementary Fig. S4). This makes MS1FA especially useful for microbial metabolomics and natural product research, where reference MS2 data for secondary metabolites are often unavailable.

To evaluate the performance in terms of feature annotation and correct feature grouping of MS1FA, we generated a second dataset by spiking sixteen natural products into a complex *P. aeruginosa* metabolome extract (StM16). These compounds were selected because they tend to produce multiple MS1 features. To establish a reliable ground truth, we acquired reference spectra of the 16 standards across the full mass range, then cleaned and subtracted background signals, thereby defining the set of features derived from each individual compound. The MS1 signals of the reference spectra provided a reference against which the performance of the feature grouping of MS1FA in comparison to CAMERA and MZmine4 was evaluated. By this means False Discovery Rate (FDR) and True Positive Rate (TPR) and the respective confusion matrices could be calculated (Supplementary Tables S4 and S5 and Supplementary Figs S5–S7). MS1FA achieved the lowest FDR of 0.031 for feature grouping by intersecting “group” and “corgroup” assignments. Utilizing the union of the grouping method increased the TPR to 0.947 (at the cost of a higher FDR). In comparison, CAMERA and MZmine4 exhibited higher FDR of 0.155 and 0.152, respectively (Supplementary Table S5). Feature annotation was assessed by the number of correctly identified adducts, neutral losses, isotopes, and in-source fragments, as detailed in Supplementary Tables S7 and S8. Overall, MS1FA was able to annotate more features correctly compared to CAMERA, MZmine4 and ISFrag (Supplementary Tables S1 and S2).

In summary, MS1FA provides some unique capabilities such as feature grouping by any relation (Supplementary Table S6). In addition, it offers a unique combination of functionalities as well as the ease of use and interactivity of a web-based platform. The dual group indices assist users in refining their interpretation of annotation results and in double-checking their accuracy, leading to more confident metabolite identification. Users should be aware that false-positive matches can still lead to misannotations. This risk is particularly relevant when isomeric compounds with overlapping retention times and similar MS2 spectra are incorrectly assigned to the same feature group.

## 4 Conclusion

MS1FA is an interactive web-based Shiny app that enables comprehensive MS1 feature annotation of untargeted metabolomics datasets. It uniquely combines methods for identifying and annotating redundant features, which often presents a major challenge in the analysis. MS1FA processes data from widely used tools such as XCMS and MZmine, outputting an annotated feature table that can be interactively explored and exported. Key features include the matching of MS1 features to MS2 fragment data and correlation network analysis of intensity distributions post-perturbation, which are crucial for identifying in-source fragments. The app also annotates neutral losses, isotopes, adducts, and multimers, and supports metabolite identification through target list matching. Interactive plots allow users to explore correlation networks and intensity profiles, which are linked to the annotated peak table, providing an additional layer of data exploration.

## Supplementary Material

btaf161_Supplementary_Data

## Data Availability

The metabolomics datasets are available in the MassIVE repository under accession numbers MSV000097219 and MSV000086820 (https://massive.ucsd.edu/).
