# Supplementary material for: MS1FA: Shiny app for the annotation of redundant features in untargeted metabolomics datasets
Source: Bioinformatics. 2025 Apr 15;41(5):btaf161. doi: 10.1093/bioinformatics/btaf161 (PMC12069231; doi:10.1093/bioinformatics/btaf161)
Supplement: btaf161_Supplementary_Data [file btaf161_supplementary_data.zip › Supplementary_Information_final.docx]

**Supplementary Information**

MS1FA: Shiny App for the Annotation of Redundant Features in Untargeted Metabolomics Datasets

Ruibing Shi^1^, Frank Klawonn^1,2^, Mark Brönstrup^3,4^ and Raimo Franke^3,*^

1 Biostatistics Research Group, Helmholtz Centre for Infection Research, Braunschweig, Germany

2 Department of Computer Science, Ostfalia University of Applied Sciences, Wolfenbuettel, Germany

3 Department of Chemical Biology, Helmholtz Centre for Infection Research, Braunschweig, Germany

4 German Centre for Infection Research (DZIF), partner site Hannover-Braunschweig, Germany

* Author to whom correspondence should be addressed:

Dr. Raimo Franke

Email: raimo.franke@helmholtz-hzi.de

Table of Contents

[1 Experimental methods 3](#_Toc191545764)

[1.1 LC-MS/MS measurements of StM16 standards mixture spiked into a *Pseudomonas aeruginosa* PA14 extract 3](#_Toc191545765)

[1.2 LC-MS/MS measurements of antibiotics perturbed *Pseudomonas aeruginosa* PA14 3](#_Toc191545766)

[2 Description of key concepts 4](#_Toc191545767)

[2.1 Underlying concept for the implementation of correlation groups. 4](#_Toc191545768)

[2.2 Annotation of in source fragments (ISFs) using MS2 data 5](#_Toc191545769)

[2.3 Grouping of related features 7](#_Toc191545770)

[3 Evaluation of MS1FA in comparison to other tools 9](#_Toc191545771)

[3.1 Evaluation of feature annotation 9](#_Toc191545772)

[3.2 Evaluation of feature grouping 10](#_Toc191545773)

[4 Functionality comparison with other tools 16](#_Toc191545774)

[5 Case study 17](#_Toc191545775)

[6 References 22](#_Toc191545776)

# Experimental methods

## LC-MS/MS measurements of StM16 standards mixture spiked into a *Pseudomonas aeruginosa* PA14 extract

**Reagents**. Chemical standards of the StM16-Mix were obtained from Sigma-Aldrich. Acetonitrile Ultra LC/MS grade and water Ultra LC/MS grade were obtained from J.T. Baker.

**Preparation of PA14 extract spiked with StM16-Mix at different concentrations.** A mixture of 16 compounds - 2-methoxybenzoic acid, biochanin A, trans-ferulic acid, 3-indoleacetonitrile, indole-3-carboxaldehyde, kinetin, p-coumaric acid, L-(+)-α-phenylglycine, phloridzin dihydrate, rutin trihydrate, indole-3-acetyl-L-valine, quercetin, naproxen, nortriptyline, trimethoprim, and novobiocin - was prepared at a concentration of 120 µM per compound in a 1:1 mixture of acetonitrile and water.

The StM16-Mix was then spiked into a *Pseudomonas aeruginosa* PA14 extract, prepared according to Franke et al. (mSystems, 2021), to achieve final concentrations of 10, 8, and 4 µM.

**LC-MS/MS analysis.** For each concentration, 1 µl of the StM16-spiked PA14 extract was analyzed by reversed-phase ultra-high-performance liquid chromatography coupled to quadrupole time-of-flight mass spectrometry with n= 3 technical replicates. Sample separation was performed on a UltiMate 3000 UPLC system (Thermo Fisher Scientific, Dreieich, Germany) using a 150 by 2.1 mm Kinetex C18 column with 1.7 µm particle size (Phenomenex, Aschaffenburg, Germany) with a flow rate of 300 µl/min; gradient elution with water with 0.1% (vol/vol) formic acid as eluent A and acetonitrile with 0.1% (vol/vol) formic acid as eluent B was run as follows: 1% B from t = 0 min to t = 2 min, linear gradient from 1% B to 100% B from t = 2 min to t = 20 min, hold 100% B until t = 25 min, and return to start conditions with 1% B, overall runtime was 30 min.

The samples were analyzed by positive mode electrospray ionization quadrupole time-of-flight mass spectrometry on a maXis HD QTOF (Bruker, Bremen, Germany) in full scan mode (50 to 1,500 Da). Accurate masses were obtained by internal calibration using an ion cluster of sodium formate and lock mass calibration. The StM16-Mix was also analyzed using data-dependent MS/MS by collision-induced dissociation of the five most abundant ions in each scan, making use of the Bruker smart exclusion algorithm.

The raw data of the LC-MS/MS measurements were deposited as MassIVE data set MSV000097219 (https://massive.ucsd.edu/).

## LC-MS/MS measurements of antibiotics perturbed *Pseudomonas aeruginosa* PA14

Experimental procedures and LC-MS/MS conditions are described in Franke et al. (2021). The raw LC-MS/MS data in mzXML format are available in the GNPS/MASSIVE repository under accession number MSV000086820.

# Description of key concepts

## Underlying concept for the implementation of correlation groups

The challenges for untargeted microbial metabolomics datasets have arisen due to several reasons: microbes can produce a wide variety of (secondary) metabolites, which may not be present in current databases. Spectral databases may be non-existent or incomplete for a particular organism and the lack of reference standards and complex sample matrices further complicate the analysis. Grouping of redundant peaks that are derived from the same metabolite can be achieved by using their differential abundance.

The idea is that redundant peaks derived from the same metabolite should co-vary from condition to condition. Our novel tool MS1FA uses correlation of feature abundance patterns that can be induced by deliberate perturbations of the microbial metabolomes, a method we coined “annotation by perturbations” (Figure S1). It shows that peaks that correspond to ions derived from the same metabolite experience a similar reduction upon perturbation, whereas background peaks remain unaffected. The right figure shows exemplary box plots of the intensity distributions of L-phenylalanine [M+H]^+^ ion and one of its in-source fragments, illustrating a clear correlation of the intensity patterns of the two features.


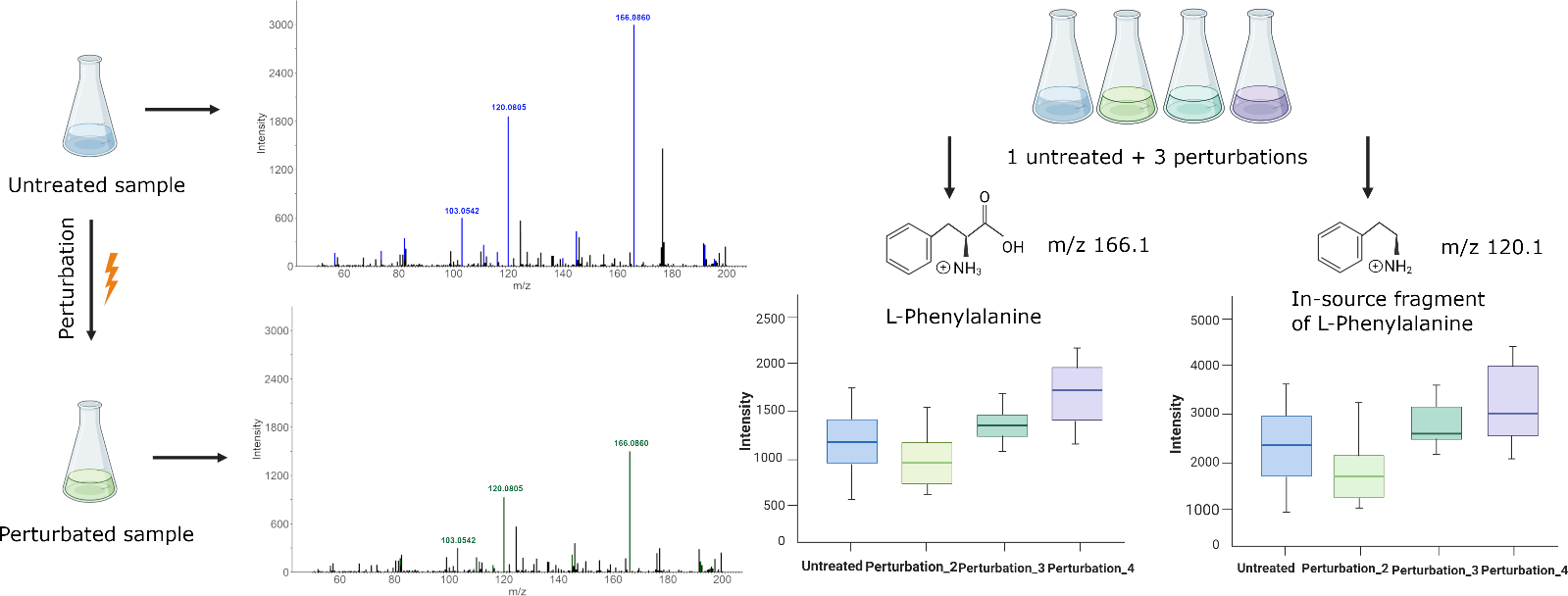


Figure S1. Generation of feature abundance patterns through metabolome perturbation to aid feature annotation. The left part of the figure shows the mass spectra of an untreated and a perturbed sample. The intensities of the peaks that correspond to ions that are derived from the same metabolite experience a similar reduction upon perturbation, whereas background peaks remain unaffected. The right part of the figure shows exemplary box plots of the intensity distributions of L-phenylalanine [M+H]^+^ ion and one of its in-source fragments, illustrating a clear correlation of the intensity patterns of the two ions.

**Important Consideration**

Our correlation-based method assumes that in-source fragmentation efficiencies remain consistent and that experimental conditions (e.g., growth conditions, sample matrix) do not vary so drastically as to alter ionization behavior in unpredictable ways. Researchers should ensure stable LC-MS settings and consider normalizing for differences in biomass or sample load. Where drastic changes in sample matrix or growth conditions are expected, the correlation method might not perform as intended, and users should consider using our alternative grouping method (“grouping of related features”).

## Annotation of in source fragments (ISFs) using MS2 data

In-source fragmentation in the ESI ion source is a significant factor contributing to feature redundancies, complicating the analysis of untargeted metabolomics datasets. When an in-source fragment of one metabolite matches the [M+H]^+^ ion of another, misannotation is likely to occur, particularly in short gradients or direct infusion analyses. Notably, in-source fragments often correspond closely to fragment ion peaks in the MS2 spectrum of the same metabolite, especially at low collision energies.

MS1FA can utilize MS2 data from either a single mzXML or mzML file, such as one generated from a pooled sample measurement, or from an MGF file compiled from multiple MS2 files using MZmine. MS1FA matches precursor ions from the MS2 data to MS1 features in the feature table. It then searches the feature table within a defined time window around the matched precursor ion mass to find any MS2 fragment ions that correspond to features, using a user-defined mass accuracy threshold. If an MS2 fragment ion matches a feature, that feature is annotated in the "**ISF_anno**" column of the feature table. This process links the MS2 data to the corresponding MS1 features.

Figure S2 illustrates these ideas for the metabolite L-phenylalanine: the precursor of the MS2 spectrum of m/z = 166.0862 that was measured at 196.427 seconds can be matched to feature FT0252 in the feature table with m/z = 166.0860, corresponding to the [M+H]^+^ ion of phenylalanine. Thus, it gets the entry “precursor” in the column “ISF annotation”. All fragment ions in the spectrum are attempted to be matched with features in the feature table. Figure S2 shows that the fragment ions with m/z = 120.0807 and 103.0541 can be matched to features FT0253 and FT0255 in the feature table, which consequently get the annotation “MS2_match”, supporting the hypothesis that FT0253 and FT0255 are in-source fragments of phenylalanine.

The annotation process becomes more complex when fragment ions themselves act as precursors in MS2 spectra generated in DDA mode. Figure S3 displays a screenshot of the MS1FA feature table, filtered to show only the group of ions originating from phenylalanine, with their corresponding annotations, as also presented in Table S1. The [M+H]^+^ ion with m/z 166.0860 is given the additional label 'FT0252_MS2 match,' indicating that this m/z value also appears as a peak in the MS2 spectrum. This is not always the case, especially at higher collision energies, where the precursor may no longer be present as a peak in the MS2 spectrum. The ion corresponding to m/z 120.0805, labeled as FT0253, receives additional labels 'Precursor' and 'FT0253_MS2 match' because it acts as a precursor itself and appears as a peak in its own MS2 spectrum, hence the label 'FT0253_MS2 match.'


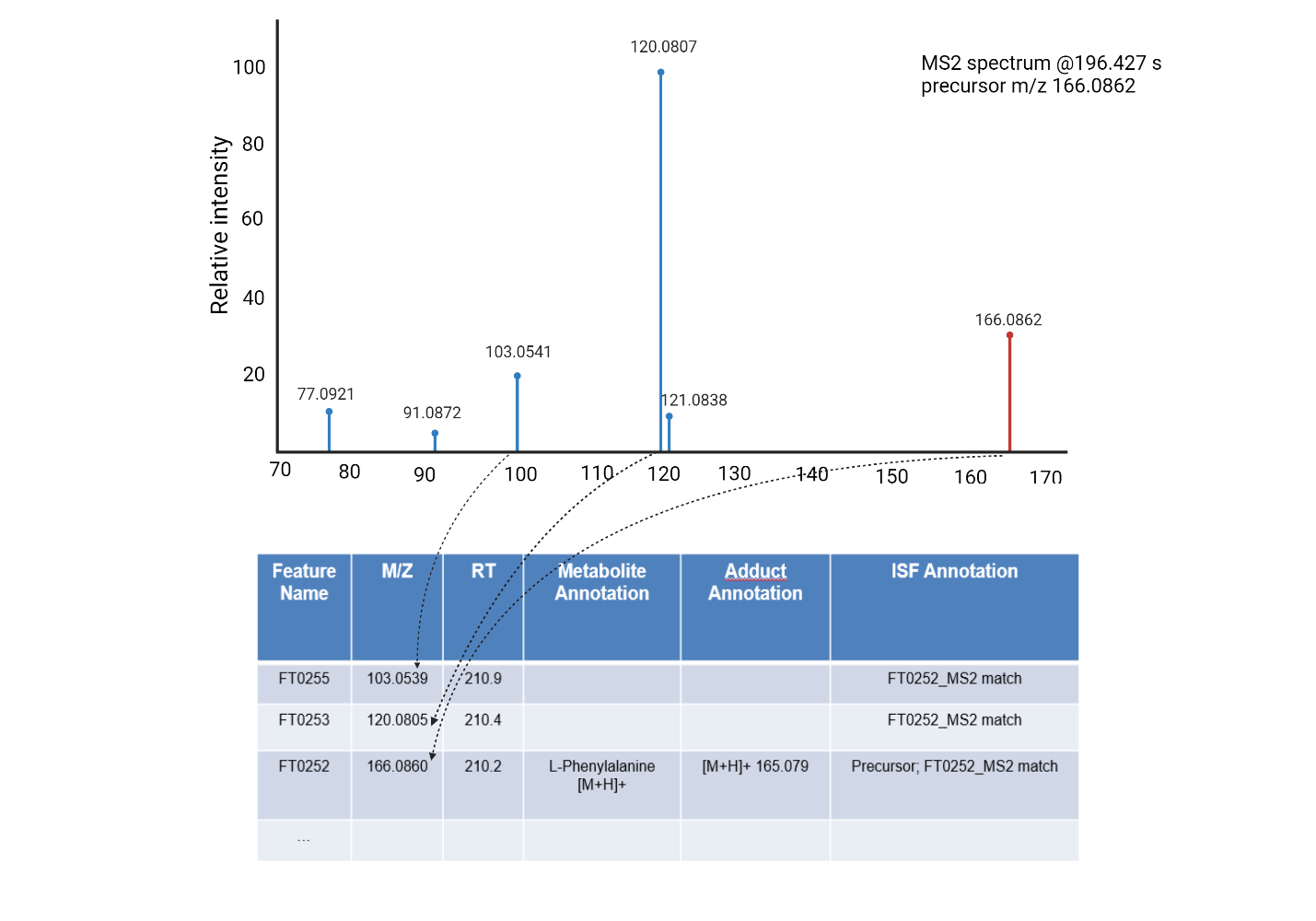


Figure S2. Scheme of ISF feature annotation by matching MS2 data (see spectrum) to MS1 features given in the table.

## Grouping of related features

This grouping method consolidates features that have any relationship with each other -such as in-source fragments, adducts, neutral losses, and isotopes- into a single group, ensuring comprehensive capture of related features. The method works independently of correlation-based grouping and provides valuable assistance in feature annotation and metabolite identification when strong correlation patterns are absent. Additionally, the group index and correlation group index can mutually verify the annotation of redundant features. As demonstrated with L-phenylalanine in Figure S3, all redundant features are correctly annotated and grouped together by both grouping methods. Figure S4 shows the correct annotation of 27 features, all originating from the metabolite phlorizin. Both grouping methods are in agreement, and the correct annotation of the neutral loss of anhydrohexose even provides structural information.


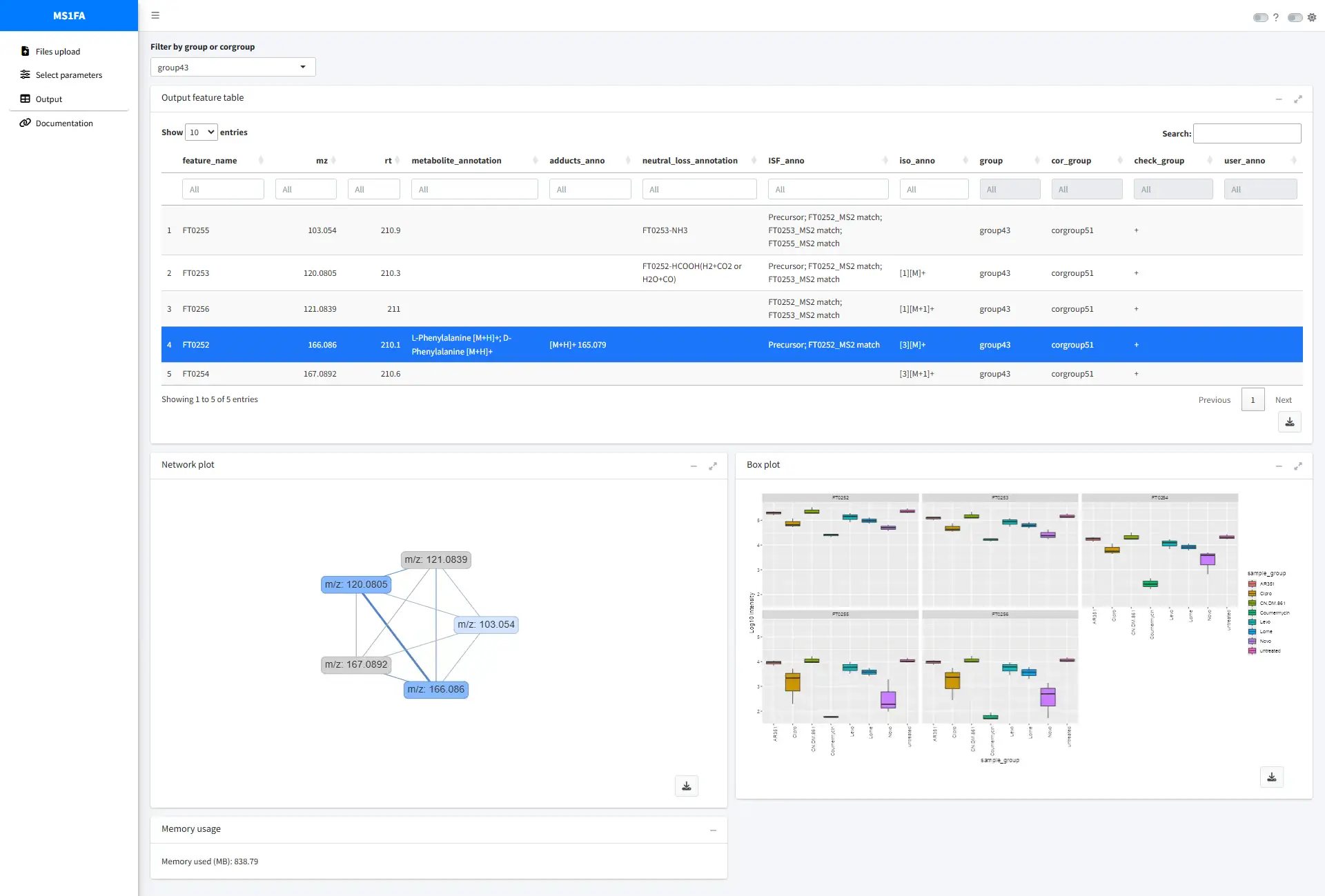


Figure S3. Screenshot of MS1FA output of L-phenylalanine and its redundant features.

Chemical Structure of phlorizin, a glucoside of phloretin.

Chemical Formula: C_21_H_24_O_10_

Exact Mass: 436.1369

[M+H]^+^ m/z: 437.1443


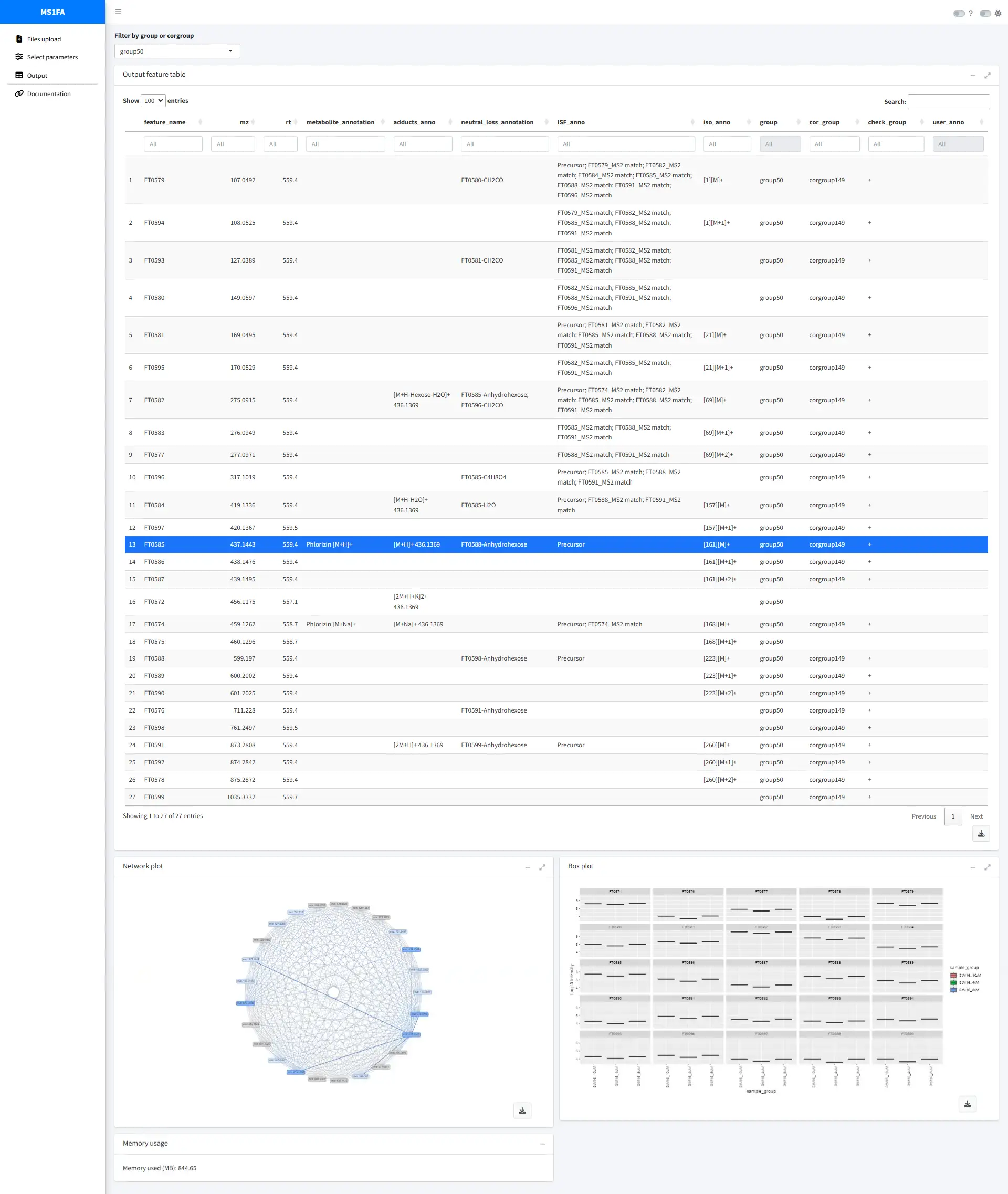


Figure S4. Chemical structure of phlorizin and filtered feature table of the StM16 analysis.

Figure S4 shows the peak table of the StM16 dataset, filtered by “group50”. The metabolite giving rise to these features could be identified as phlorizin (FT0585), a glucoside of phloretin. The neutral loss of anhydrohexose as in FT0582 was correctly annotated by MS1FA, demonstrating that even structural information can be derived from the MS1FA annotation.

# Evaluation of MS1FA in comparison to other tools

## 3.1 Evaluation of feature annotation

We compared the feature annotation accuracy of MS1FA to MZmine 4, CAMERA and ISFrag. To ensure a fair comparison, we examined both MZmine’s "Best Ion" and "row identity all IDs" annotations. Since MZmine automatically removes isotopic peaks, its feature table does not include isotope entries. CAMERA records isotopic features in the "isotopes" column, while adducts and neutral losses are listed under "adduct." Notably, we only considered adduct annotations when they refer to the correct neutral mass (M).

| Sample | Peak-picking software | Identified metabolites | Annotation tool | Overall  adduct  annotation | Isotopes/multiple charged ions | Overall neutral loss annotation | Overall in-source fragment  annotation | Sum of  annotated features |
| --- | --- | --- | --- | --- | --- | --- | --- | --- |
| PA14 | MZmine4 | 58 | MS1FA | 103 | 0 | 52 | 37 | 192 |
|  |  |  | MZmine4 | 90 | - | 10 | - | 100 |
|  | XCMS | 58 | MS1FA | 112 | 80 | 32 | 34 | 258 |
|  |  |  | CAMERA | 67 | 70 | 10 | - | 147 |
|  |  |  | ISFrag | - | - | - | 10 | 10 |

Table S1. Comparison of feature annotations for the PA14 dataset by MS1FA vs. MZmine4 and XCMS-CAMERA, XCMS-ISFrag.

| Sample | Peak-picking software | Identified metabolites | Annotation tool | Overall  adduct  annotation | Isotopes/multiple charged ions | Overall neutral loss annotation | Overall in-source fragments  annotation | Sum of  annotated features |
| --- | --- | --- | --- | --- | --- | --- | --- | --- |
| StM16 | MZmine4 | 16 | MS1FA | 68 | 7 | 79 | 141 | 295 |
|  |  |  | MZmine4 | 50 | - | 5 | - | 55 |
|  | XCMS | 16 | MS1FA | 66 | 154 | 69 | 178 | 467 |
|  |  |  | CAMERA | 71 | 154 | 13 | - | 238 |
|  |  |  | ISFrag | - | - | - | 109 | 109 |

Table S2. Comparison of feature annotations for StM16 with 16 standards spiked into a *Pseudomonas aeruginosa* PA14 extract by MS1FA vs. MZmine4 and XCMS-CAMERA, XCMS-ISFrag.

## 3.2 Evaluation of feature grouping

To assess the accuracy of feature grouping, we analyzed the StM16 dataset, which includes 16 standards spiked into a *Pseudomonas aeruginosa* PA14 extract used as a complex matrix. The standard compounds were selected because they tend to produce multiple MS1 features. To establish a reliable ground truth, we acquired reference spectra of the 16 standards across the full mass range, then cleaned and subtracted background signals, thereby defining the set of features derived from each individual compound. The reference spectra are stored in the Si16.library file, which has been deposited in the GitHub repository. The MS1 signals of the reference spectra provided a reference against which the performance of feature grouping by MS1FA in comparison to CAMERA and MZmine4 was evaluated. By this means, the False Discovery Rate (FDR) and True Positive Rate (TPR) and the respective confusion matrices could be calculated. To identify features derived from each reference compound, primary ion m/z values from Table S3 were first matched to MZmine and XCMS feature tables based on exact mass and a retention time window of ±3 seconds to the reference RT in Table S3. Subsequently, neighboring features within ±3 seconds of the primary ion features were compared to the m/z values in the reference spectra of the StM16 compounds library, using a 0.01 m/z tolerance.

For each group of features, we determined the True Positives (TP), False Negatives (FN), False Positives (FP), and True Negatives (TN) to enable subsequent calculations of False Discovery Rate (FDR) and True Positive Rate (TPR). Detailed definitions are in Table S4.

| Compound name | Formula | Adduct | m/z | RT (seconds) |
| --- | --- | --- | --- | --- |
| 2-Methoxybenzoic Acid | C_8_H_8_O_3_ | [M+H]^+^ | 153.0546 | 506.171 |
| 3-Indoleacetonitrile | C_10_H_8_N_2_ | [M+H]^+^ | 157.0760 | 635.685 |
| Biochanin A | C_16_H_12_O_5_ | [M+H]^+^ | 285.0757 | 794.759 |
| Indole-3-acetyl-L-valine | C_15_H_18_N_2_O_3_ | [M+H]^+^ | 275.1390 | 623.672 |
| Indole-3-carboxaldehyde | C_9_H_7_NO | [M+H]^+^ | 146.0600 | 536.371 |
| Kinetin | C_10_H_9_N_5_O | [M+H]^+^ | 216.0879 | 417.541 |
| L-(+)-Alpha-Phenylglycine | C_8_H_9_NO_2_ | [M+H]^+^ | 152.0706 | 85.7752 |
| Naproxen | C_14_H_14_O_3_ | [M+H]^+^ | 231.1016 | 750.693 |
| Nortriptylin | C_19_H_21_N | [M+H]^+^ | 264.1747 | 679.889 |
| Novobiocin | C_31_H_36_N_2_O_11_ | [M+H]^+^ | 613.2391 | 916.383 |
| p-Coumaric Acid | C_9_H_8_O_3_ | [M+H]^+^ | 165.0546 | 489.72 |
| Phlorizin | C_21_H_24_O_10_ | [M+H]^+^ | 437.1442 | 559.138 |
| Quercetin | C_15_H_10_O_7_ | [M+H]^+^ | 303.0499 | 512.035 |
| Rutin | C_27_H_30_O_16_ | [M+H]^+^ | 611.1607 | 512.035 |
| Trans-Ferulic Acid | C_10_H_10_O_4_ | [M+H]^+^ | 195.0652 | 515.561 |
| Trimetoprim | C_14_H_18_N_4_O_3_ | [M+H]^+^ | 291.1452 | 445.785 |

Table S3. List of the sixteen spiked compounds (StM16).

| Scenario | Classification | Details |
| --- | --- | --- |
| True peaks correctly grouped | True Positives (TP) | Peaks in the feature table that match peaks in the reference spectrum within the defined tolerance. |
| True peaks missing in the group | False Negatives (FN) | Peaks present in the reference spectrum but incorrectly grouped in the feature table. |
| Peaks in the group but not in the library. | False Positives (FP) | Features grouped in the feature table that do not match any peaks in the reference spectrum. |
| Peaks not in the group and not in the library peaks. | True negatives (TN) | Peaks in the feature table that are neither grouped nor correspond to any peaks in the reference spectrum. |

Table S4. Definition of True Positives (TP), False Negatives (FN), False Positives (FP) and True negatives (TN)

In MS1FA, we introduced two grouping methods: "group" and "corgroup". The "group" method merges related features, such as in-source fragments, adducts, neutral losses, and isotopes, while "corgroup" clusters features based on intensity pattern correlation. To assess consistency between these methods, we implemented an additional "check group" column that pairs "group" and "corgroup" to identify the largest intersection of the two group assignments. When a match is found, a "+" is assigned to indicate agreement. A correct assignment in both methods strengthens annotation confidence.

To compare the grouping performance, we compared **MS1FA ("group" and "corgroup")**, **MZmine ("correlation group ID")**, and **CAMERA ("pcgroup")** using a **confusion matrix**, calculating the **False Discovery Rate (FDR)** and **True Positive Rate (TPR)** based on the processing of the 16 spiked standards (StM16). For MS1FA, features were further categorized into:

1. Present in either "group" or "corgroup"
2. Present in both "group" and "corgroup"
3. Only in "group"
4. Only in "corgroup"

| Processing software | MZmine | | XCMS | |
| --- | --- | --- | --- | --- |
| Evaluation metrics | **FDR** | **TPR** | **FDR** | **TPR** |
| MS1FA: group Or corgroup | 0.119 | **0.906** | 0.091 | **0.947** |
| MS1FA: group And corgroup | **0.063** | **0.600** | **0.031** | **0.679** |
| MS1FA: group | 0.100 | 0.740 | 0.083 | 0.813 |
| MS1FA: corgroup | 0.094 | 0.766 | 0.051 | 0.813 |
| MZmine correlation group | **0.152** | 0.765 | - | - |
| CAMERA pcgroup | - | - | **0.155** | 0.935 |

Table S5. False Discovery Rate (FDR) and True Positive Rate (TPR) for feature grouping methods across MS1FA, MZmine and CAMERA. Higher TPR indicates better sensitivity in detecting true feature relationships, while lower FDR means fewer false positive groupings.

| 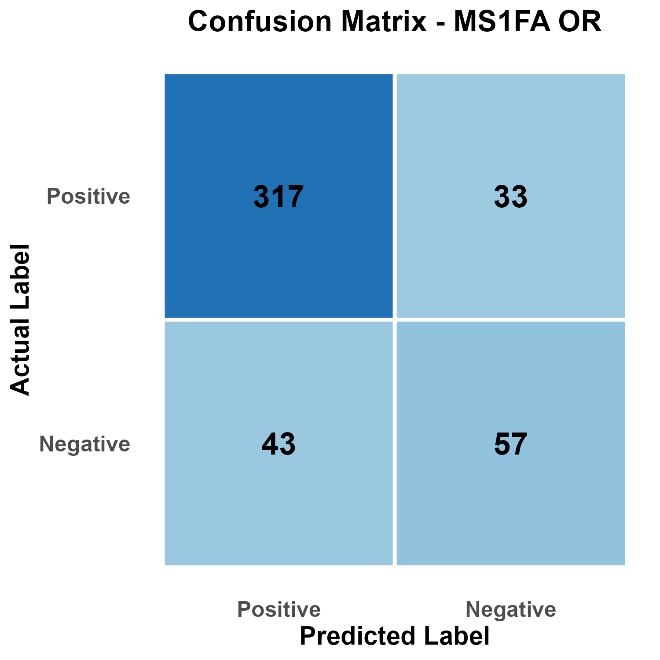  B)  A)  C) | 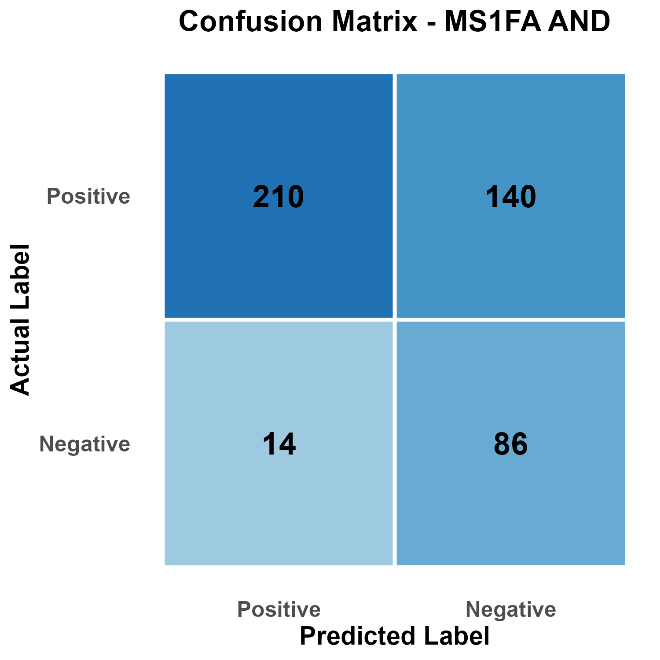  D) |
| --- | --- |
| 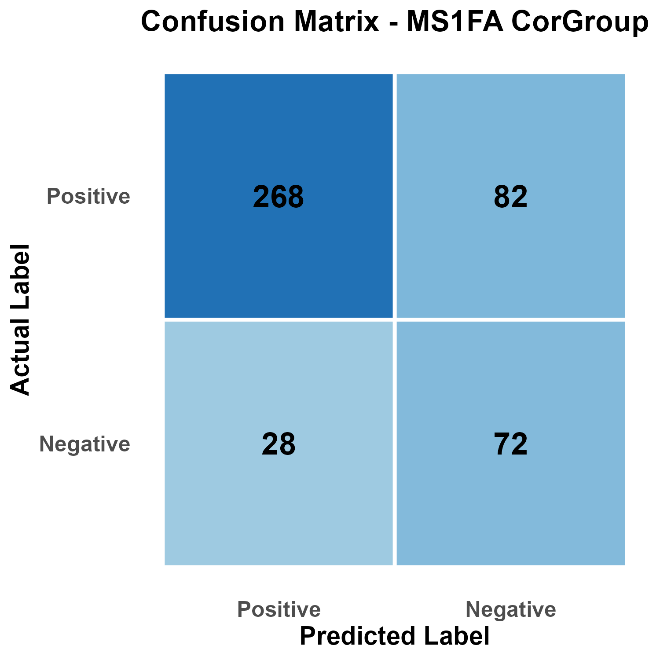 | 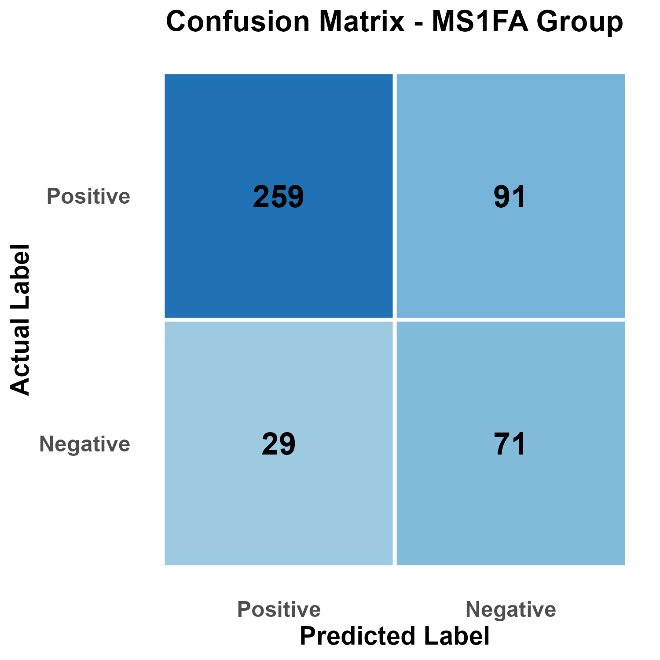 |

Figure S5. Confusion matrices of MS1FA grouping using the MZmine feature table with the number of True Positives (TP), False Positives (FP), False Negatives (FN), and True Negatives (TN). A) MS1FA features grouped by “corgroup” or “group” B) MS1FA features grouped by “corgroup” and “group” C) MS1FA features grouped by “corgroup” D) MS1FA features grouped by “group”.

| 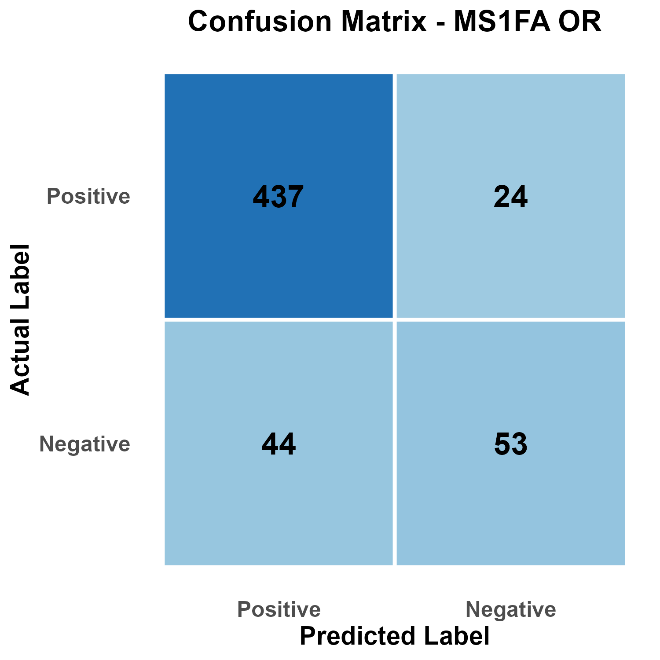 | 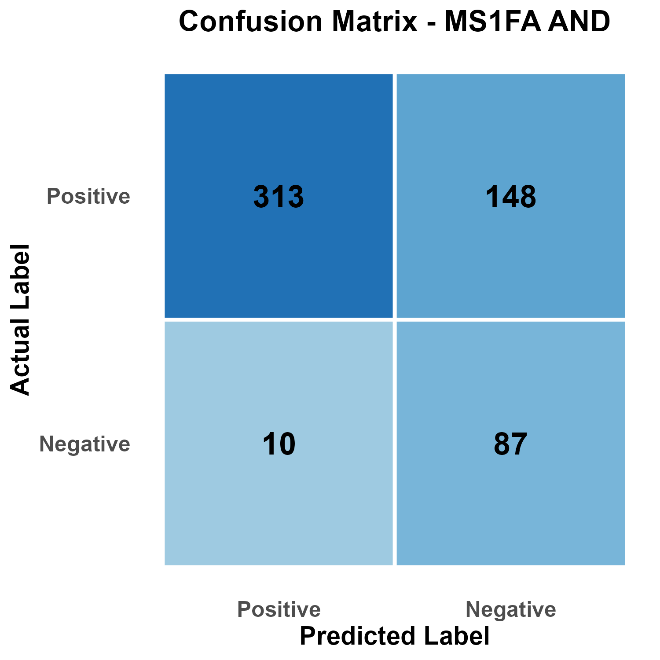  D) |
| --- | --- |
| 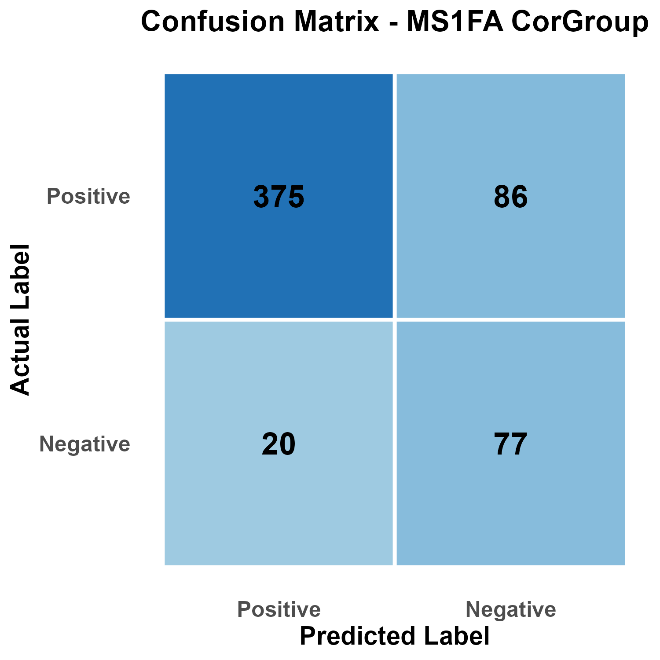  C) | 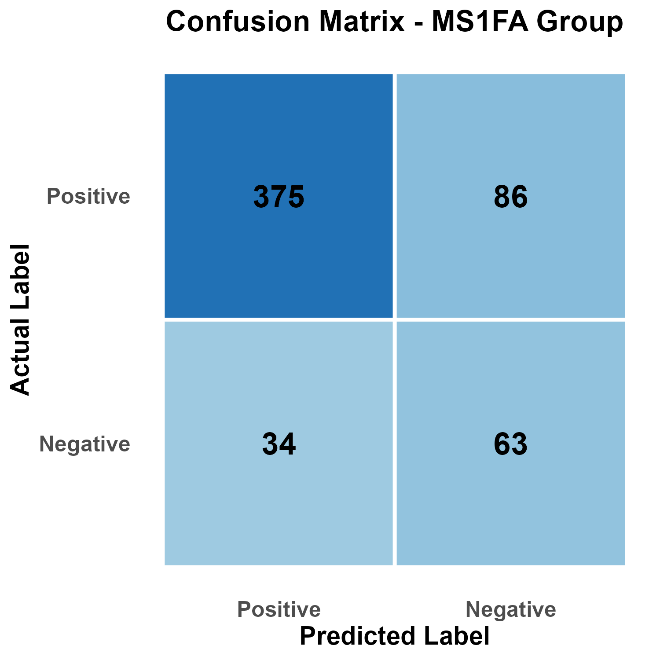 |

Figure S6. Confusion matrices of MS1FA grouping using the XCMS feature table with the number of True Positives (TP), False Positives (FP), False Negatives (FN), and True Negatives (TN). A) MS1FA features grouped by “corgroup” or “group” B) MS1FA features grouped by “corgroup” and “group” C) MS1FA features grouped by “corgroup” D) MS1FA features grouped by “group”.

A)

B)

| 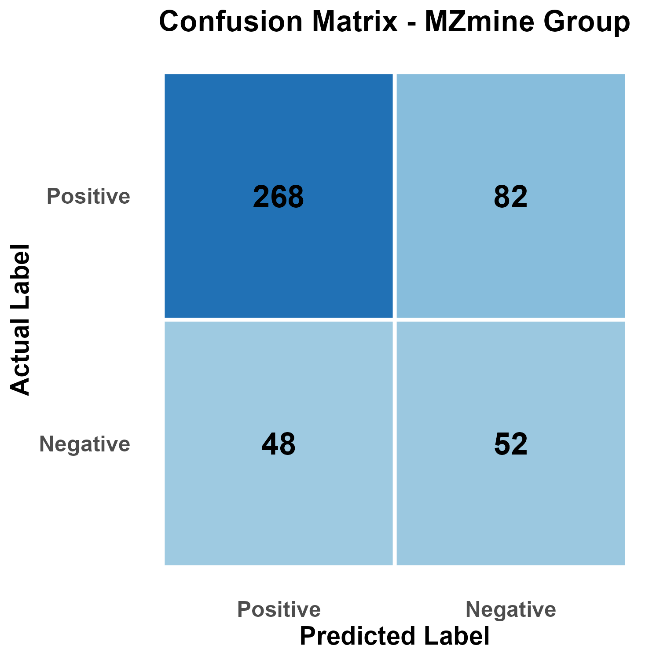 | 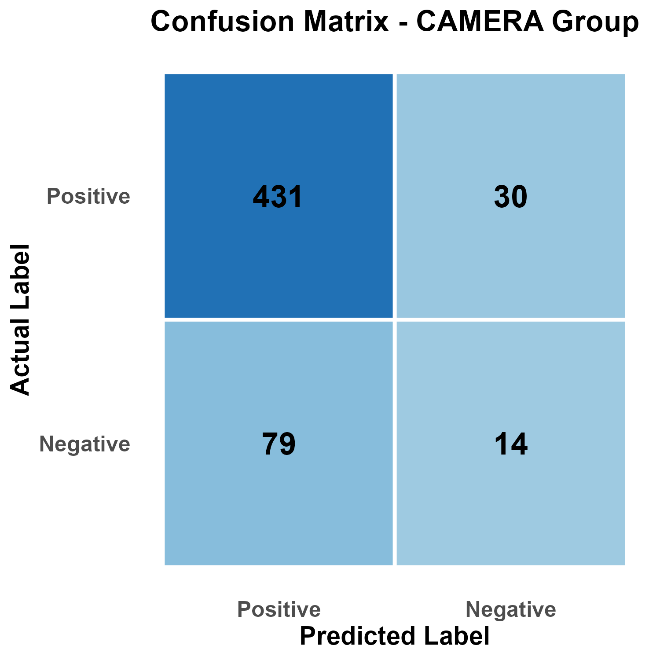 |
| --- | --- |

Figure S7. Confusion matrices of MZmine grouping and CAMERA with the number of True Positives (TP), False Positives (FP), False Negatives (FN), and True Negatives (TN). A) Confusion matrix of MZmine grouping “correlation group” B) Confusion matrix of CAMERA “pcgroup”.

A)

B)

# Functionality comparison with other tools

Table S6 compares MS1FA with three existing tools. MS1FA’s key advantage is its integration of all major annotation approaches for redundant features in a single interactive platform. It combines correlation-based grouping with effective and reliable ISF annotation using MS2 data. MS1FA can work with MS1 data, MS2 data, or a combination of both, and its grouping method based on feature relationships is also distinct. Additionally, it is the only web-based platform offering these comprehensive annotation capabilities, ensuring easy accessibility. Users can explore the annotated feature table interactively with correlation networks and intensity distribution plots, annotate features, and download the results.

| **Functionalities** | **MS1FA** | **MZmine** | **CAMERA** | **ISFrag** |
| --- | --- | --- | --- | --- |
| 1.  Metabolite identification by exact mass match | + | + | - | + |
| 2. Adduct annotation | + | + | + | - |
| 3. Neutral loss annotation | + | + | + | - |
| 4. ISF annotation by MS2 data | + | - | - | + |
| 5. ^13^C isotope annotation | + | + | + | - |
| 6. Grouping by intensity correlation | + | + | + | - |
| 7. Grouping by any relations | + | - | - | - |
| 8. Visualization | + | + | - | + |
| 9. Interactive usage | + | + | - | - |
| 10. Web-based platform | + | - | - | - |

Table S6. Comparison of functionalities between MS1FA, MZmine, CAMERA and ISFrag.

# Case study

Figure S8. Screenshot of MS1FA files upload.


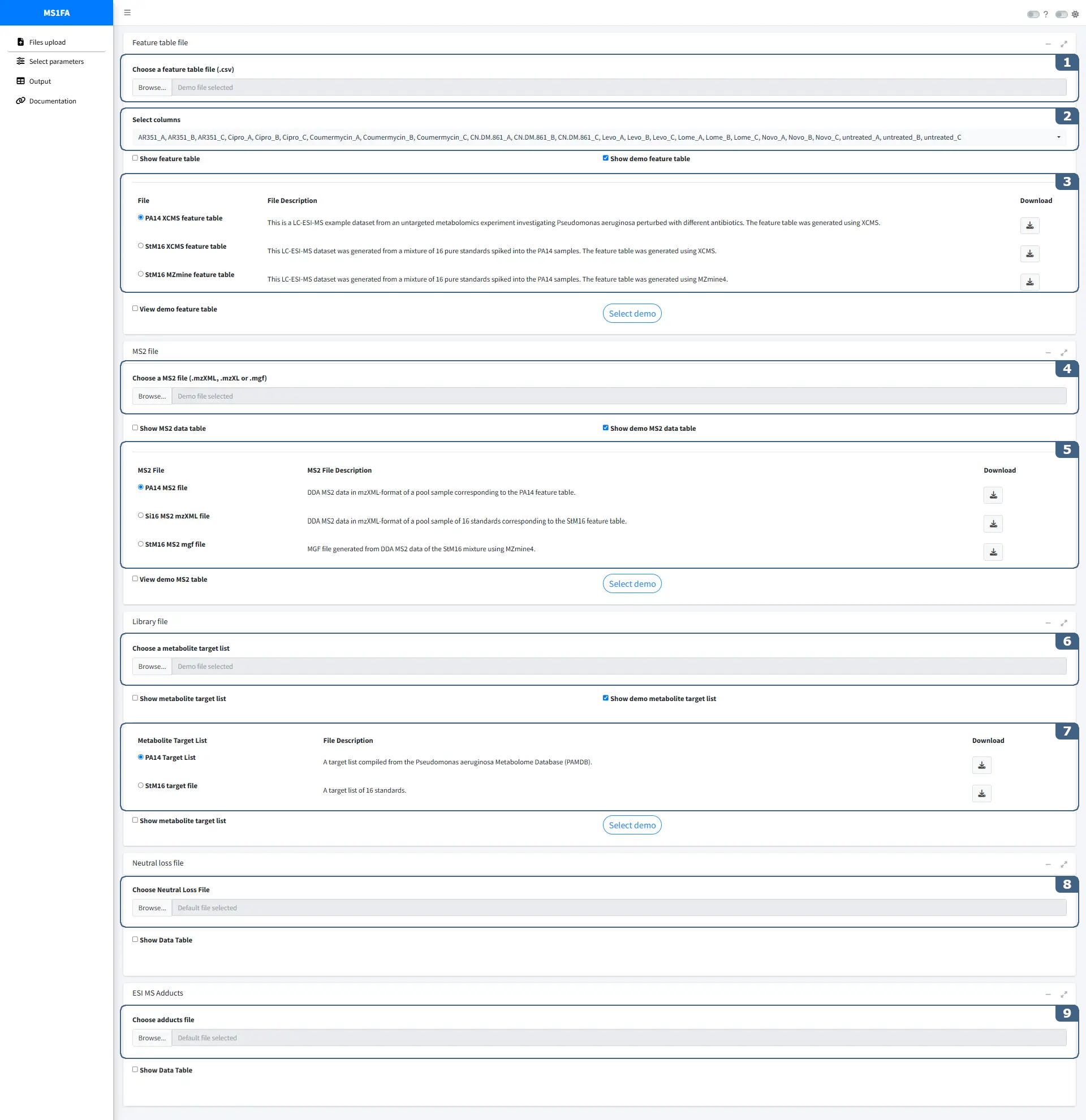


To demonstrate the functionality of MS1FA we use the provided PA14 example data. The process begins with uploading the feature table file (Section 1). Once the file is uploaded, a dropdown menu will appear (Section 2) to select the columns representing sample intensities. For replicated samples, please use consistent naming suffixes like '_A', '_B', '_C' or '_1', '_2', '_3'. In Section 3, three demo feature table files—PA14 processed via XCMS, StM16 processed via XCMS and MZmine—are available for users to test and download. Next, in Section 4, users upload an MS2 file. MS1FA supports .mzXML or .mzML files from a pool sample measured with DDA MS2, or a .mgf summary file from MZmine generated from multiple MS2 files. We provide two demo MS2 files for testing in Section 5: one is a .mzXML file for the PA14 dataset, one is a .mzXML file for the StM16 dataset, and the other is a .mgf summary file for the StM16 dataset generated from multiple MS2 files. In Section 6, users need to upload a metabolite target list in csv-format. Alternatively, it is also possible to upload spectral libraries in .msp format or Bruker .library format, which are parsed to extract the information to generate a target list. The target list must include essential information such as the metabolite name and chemical formula. In Section 7, we provide two example target lists: one for the PA14 dataset, which includes metabolites compiled from the *Pseudomonas aeruginosa* Metabolome Database (Huang et al. 2018) and a target list for the StM16 dataset, containing 16 metabolites. In section 8 and 9 default files which contain tables for neutral losses and adducts, respectively, are uploaded automatically. Upload of user-generated tables is also possible.


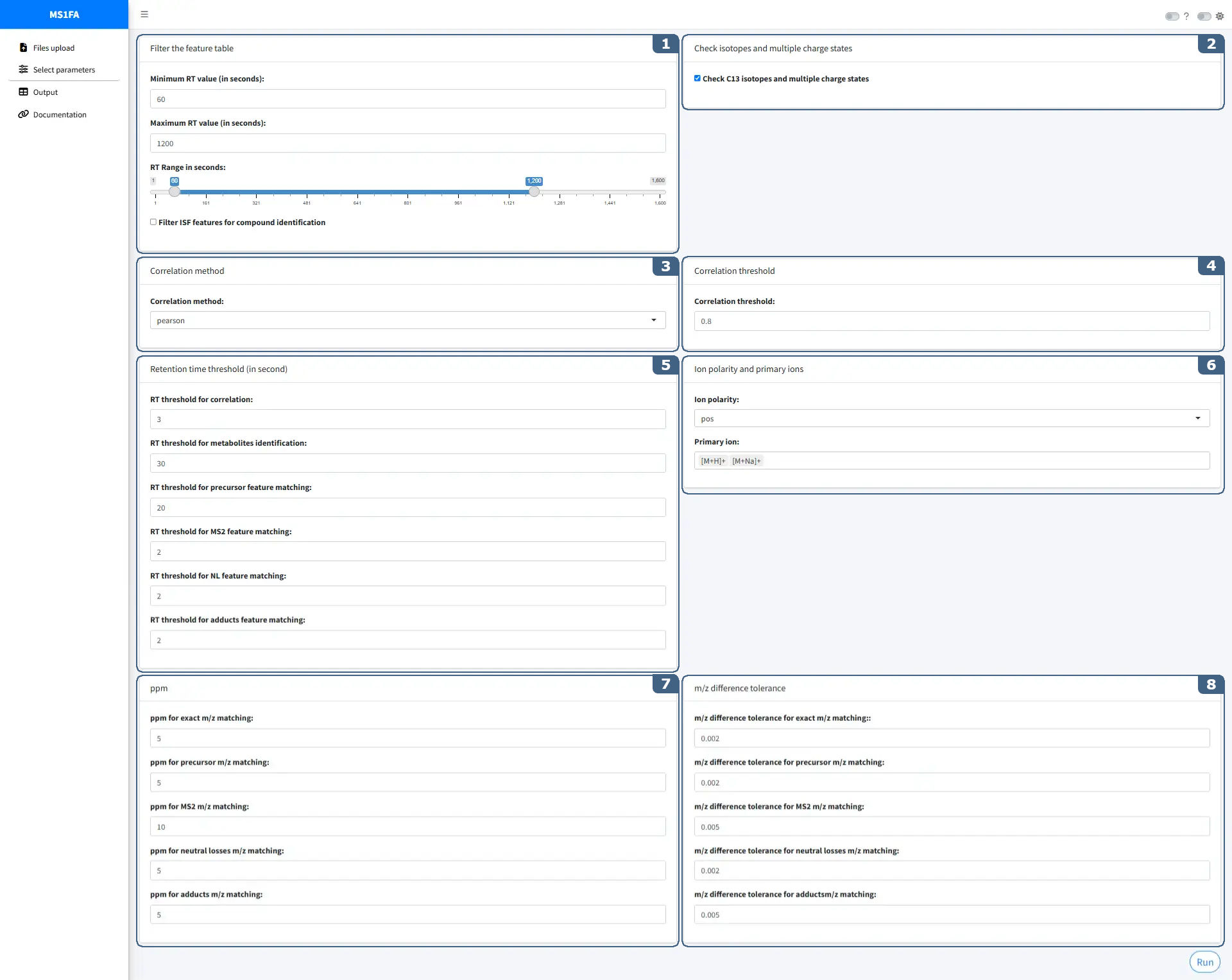


Figure S9. Screenshot of MS1FA select parameters.

Before running MS1FA, several parameters need to be set. First, the feature table is filtered by retention time (RT) to exclude unwanted features (Section 1). The default filter range, set from 60 to 1200 seconds, is designed to suit the demo datasets by removing the calibration peak and the gradient's wash phase. A checkbox is provided if the user wants to skip the metabolite annotation when the feature is already identified as a MS2 matched in-source fragment. For ^13^C isotope annotation, the checkbox is selected by default (Section 2). The next section involves choosing the measure of the correlation from the dropdown menu (Section 3) and setting the correlation coefficient threshold (Section 4), which defaults to 0.8. After that, the RT thresholds for the acceptable time windows have to be configured (Section 5): 30 seconds for metabolite identification via exact matching (if RT is provided in the target list), 20 seconds for precursor ion matching from MS2 data to the MS1 feature table, 2 seconds for matching MS2 features to precursor features in the MS1 table, and 2 seconds each for matching pairwise features to the neutral loss and adduct tables. Additionally, select the ion polarity and primary ions (Section 6), the default is positive polarity with [M+H]^+^ and [M+Na]^+^ ions, suited for the demo datasets. Next, we specify the ppm thresholds (Section 7) for exact mass matching (default 5 ppm), precursor ion m/z matching (default 5 ppm), MS2 fragment m/z matching (default 10 ppm), neutral loss m/z matching (default 5 ppm), and adduct m/z matching (default 5 ppm). Finally, we set the m/z difference tolerance thresholds (Section 8): 0.002 for exact mass matching, 0.002 for precursor ion and 0.005 for MS2 m/z matching, and 0.002 for neutral loss and 0.005 for adduct m/z matching. After setting all the parameters, click “Run” to start calculation. The default settings are well suited to process the demo data sets.


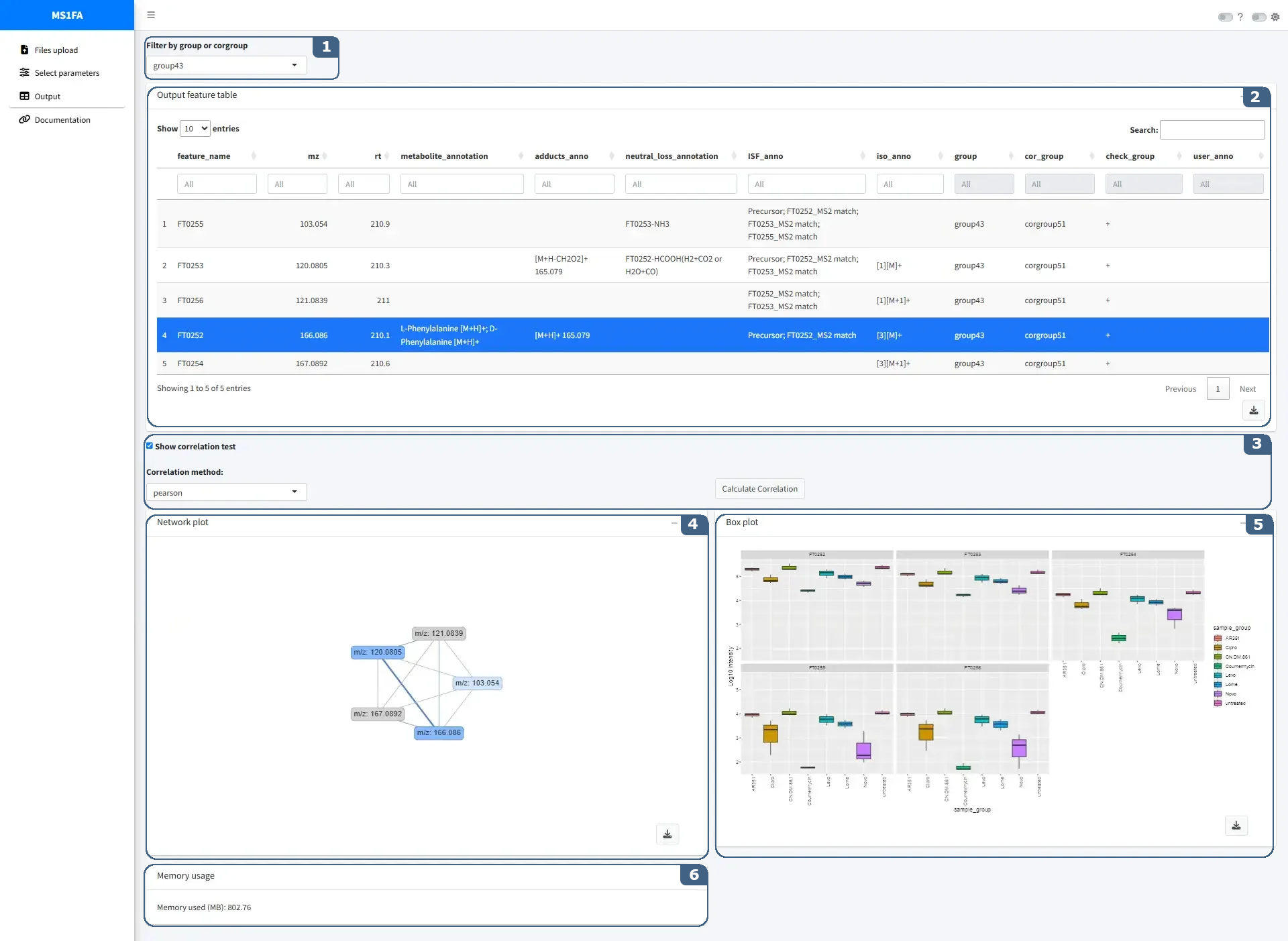


Figure S10. Screenshot of MS1FA output.

The MS1FA output includes an interactive feature table with annotations (Section 2). The first column **feature_name** contains the unique feature identifier, the second **mz** and third column **rt** display the mass-to-charge ratios and the retention time in seconds respectively. For the highlighted feature, the unique identifier is FT0252 with an m/z of 166.086 and a retention time of 210.1 seconds. The column **metabolite_annotation** shows the annotation via exact mass matching (and RT matching if available) using a target list. Matching of FT0252 to the PAMDB target list yields two matches L-Phenylalanine and D-Phenylalanine. Adducts annotation and neutral loss annotation takes place by matching to the adduct and neutral loss annotation tables, shown in the columns **adducts_anno** and **neutral_loss_annotation**, respectively. For our example, the adduct is correctly identified as [M+H]^+^ and the neutral monoisotopic mass is also calculated as 165.079. The next column shows the annotation of putative neutral losses generated by comparing each pairwise mass difference within a defined RT window to the neutral loss table. For FT0253 (m/z = 120.0805), a neutral loss of HCOOH was found in relation to FT0252 (m/z = 166.086). This neutral loss is verified in the next column **ISF_anno**, which shows the in-source-fragment (ISF) annotation via matching to MS2 data. For FT0252, we find a FT0253_MS2 match, which means that the 120.0805 m/z (FT0252) is found as a fragment ion in the MS2 spectrum with the precursor 166.086 m/z (FT0253). The column **Iso_anno** shows the annotated isotopes. The number in the first square brackets shows the isotope-group-id and [M + 1] is the first isotopic peak for the monoisotopic peak [M].The column **group** show the grouping of related features, as explained in the Supplementary Information. The results of the grouping by intensity pattern method are shown in the column **cor_group**. The column **check_group** shows “+” when the most overlapping assignment is identified by both groups. The last column **user_anno** can be used by the user to fill in comments and/or own annotations that will be exported with the annotated feature table as well.

Section 3 introduces the checkbox “Show Correlation Test”, which allows users to view correlation statistics. When exactly two rows are selected in the feature table, a dropdown menu appears, enabling the user to choose from different correlation methods. After clicking “Calculate Correlation”, a modal dialog displays the results from R’s cor.test() function.

When a row in the feature table is selected, a corresponding interactive correlation network plot is generated (Section 4), displaying feature names, annotations when hovering over nodes, and correlation coefficients along with m/z differences when hovering over edges. In the network plot, a gray node color indicates that the feature is recognized as an isotope. A dark blue node signifies that a metabolite has been annotated, while a light blue node indicates that the feature is not identified as a metabolite. Edges represent correlation values, m/z differences, and neutral loss annotations with thicker edges highlighting detected neutral losses.

Similarly, selecting a row in the feature table allows users to generate a box plot (Section 5), offering insights into the correlation related of features across different samples. The titles of each box plot is the name of feature. The classes are the samples groups and the values are the log_10_ transformed feature intensity value.

In Section 1, a dropdown menu allows filtering of the output feature table by group index or corgroup index. Section 6 shows the memory usage.

# References

Franke R, Overwin H, Häussler S *et al.* Targeting Bacterial Gyrase with Cystobactamid, Fluoroquinolone, and Aminocoumarin Antibiotics Induces Distinct Molecular Signatures in *Pseudomonas aeruginosa.* Traxler MF (ed.). *mSystems* 2021;**6**:e00610-21.

Huang W, Brewer LK, Jones JW *et al.* PAMDB: a comprehensive *Pseudomonas aeruginosa* metabolome database. *Nucleic Acids Res* 2018;**46**:D575–80.
